# Comparison of Fenestrating and Reconstituting Subtotal Cholecystectomy Techniques in Difficult Cholecystectomy

**DOI:** 10.7759/cureus.22441

**Published:** 2022-02-21

**Authors:** Ali Cihat Yildirim, Sezgin Zeren, Mehmet Fatih Ekici, Faik Yaylak, Mustafa Cem Algin, Ozlem Arik

**Affiliations:** 1 General Surgery, Kutahya Health Sciences University, Kutahya, TUR; 2 Biostatistics, Kutahya Health Sciences University, Kutahya, TUR

**Keywords:** difficult laparoscopic cholecystectomy, complication, laparoscopic cholecystectomy, biliary injury, subtotal cholecystectomy

## Abstract

Purpose

Cholecystectomy is one of the most frequently performed surgeries. Although laparoscopy is considered the gold standard approach, it cannot prevent biliary injuries. Subtotal cholecystectomy has been performed mainly to prevent biliary injuries during difficult cholecystectomies. This study aimed to analyse our subtotal cholecystectomy results for difficult cholecystectomy cases and to evaluate the fenestrating and reconstituting techniques.

Methods

Retrospective data were collected and analysed statistically for cases that underwent subtotal cholecystectomy in a single referral centre between 2015 and 2020. Comparisons were made of the patients’ age, gender, preoperative American Society of Anaesthesiologists (ASA) score, comorbidities, surgical timing, surgical procedure choice, postoperative complications, and mortality.

Results

The number of patients who underwent subtotal cholecystectomy was 46; 30.4% underwent emergent surgery and 69.6% underwent elective surgery. Twelve patients had subtotal fenestrating cholecystectomy and 34 had subtotal reconstituting cholecystectomy. Wound issues were noted in 17.4% of the patients, while 10.9% had temporary biliary fistulas that resolved spontaneously. Reoperation was performed in one patient due to high-output biliary drainage. Patients with postoperative complications had significantly higher co-morbid conditions (p=0.000), but surgery timing (p=0.192) and type of subtotal cholecystectomy (p=0.409) had no statistically significant effect on complications. Mortality showed a statistically significant correlation with patient comorbidities, surgery timing, and the type of procedure (p<0.05). Postoperative complications showed a statistically significant correlation with mortality (p<0.05).

Conclusion

Subtotal cholecystectomy prevents major biliary complications after cholecystectomy. Yet, the frequency of postoperative complications after subtotal cholecystectomy is incontrovertible. Intraoperative characteristics and the surgeon’s expertise decide the optimal choice of the subtotal cholecystectomy technique.

## Introduction

Gallstones have a prevalence of 10-15% in the general population, with some differences across countries. If acute cholecystitis is the first clinical scenario, approximately 20-40% of patients may develop biliary stone-related complications [1]. Laparoscopic cholecystectomy is the gold standard technique used on patients with symptomatic acute cholecystitis. The recommendation for these patients is to undergo an early cholecystectomy within 7-10 days of admission. This laparoscopic technique, used in surgery for over 30 years, has a range of difficulties from easy to complicated in gallbladder resections [2,3]. However, bile duct injuries are still a concern even with risk-minimising laparoscopic cholecystectomy techniques.

The safe cholecystectomy concept is based mainly on enabling a critical view of safety (CVS), as recommended by the Society of American Gastrointestinal and Endoscopic Surgeons (SAGES) [3,4]. Difficult cholecystectomy is subjective from surgeon to surgeon to some degree. But it may be described as not identifying CVS during laparoscopic cholecystectomy. However, devastating complications still occur in approximately three out of 1,000 cholecystectomies [5]. For this reason, a "less is more" technique has been advocated recently, even in referrals for hepatopancreatobiliary (HPB) surgery [6].

A possible "less is more" approach should be subtotal cholecystectomy, a century-old technique that has gone by names like partial cholecystectomy. Following the index study of Strasberg et al., who described the subtotal cholecystectomy as reconstituting and fenestrating types, it has gained more popularity and recognition as a good bail-out technique [7]. Several studies have researched the early and long-term results of subtotal cholecystectomy and its two main techniques in terms of complications and efficacy.

The present study evaluated the early results of subtotal cholecystectomy procedures for cases in emergency and elective settings. In addition to this, we also compared the two different techniques for complications and mortality.

## Materials and methods

After obtaining local ethical committee approval (the Institutional Review Board of Kutahya Health Sciences University approved this study (No. 2020/18-09)), we enrolled patients from our tertiary referral centre. These patients were over 18 years of age and were diagnosed by a physical examination and supported by radiological findings. They underwent a subtotal cholecystectomy for symptomatic cholelithiasis and acute cholecystitis between January 2015 and December 2020. This study did not share any patients’ identities or personal information. Data were collected and analysed using IBM SPSS version 20 (IBM SPSS Statistics for Windows, Version 20.0, IBM Corp, Armonk, NY, USA) included patient age, gender, preoperative American Society of Anaesthesiologists Classification (ASA) score, comorbidities, surgical timing of cholecystectomy as elective or emergency setting, surgical procedure choice (subtotal fenestrating or subtotal reconstituting cholecystectomy), documented postoperative complication, and mortality. The study omitted patients for any of the following: if they had any type of immunocompromised status, active sepsis, gallbladder cancer, pre-cholecystectomy complications after any interventional procedure like cholecystostomy, or if their cholecystectomy procedure was a part of major abdominal surgery.

The preference is to achieve a CVS during surgery. If it was not possible, the two principal techniques of subtotal cholecystectomy were performed according to the current surgical literature for difficult cholecystectomy. Laparoscopic surgery was the primary procedure used for patients who underwent fenestrating or reconstituting cholecystectomy. However, all cases in our study group underwent conversion to open surgery before performing subtotal cholecystectomy. The subtotal cholecystectomy type was chosen intraoperatively by the responsible surgeon. All patients had surgical site drains to follow possible biliary leakage. The average follow-up for all patients was approximately six months.

Statistical analysis

The statistical evaluation of the data was carried out by a 95% confidence interval with a 0.05 failure rate. Variables for quantitative data were shown as mean ± standard deviation or median (min-max), where applicable. Qualitative data were analysed with frequency tables. Contingency tables were used to examine the relationships and differences between categorical variables. The strengths of relationships between two variables were analysed using correlation coefficients. The Phi, Cramer's V, and normality coefficients were measured. A p-value of less than 0.05 was considered statistically significant. Line charts depicted the differences in postoperative complications for types of surgery.

## Results

Forty-six patients underwent subtotal cholecystectomy; 52.2% of the patients were female and 47.8% were male. The median age was 60.63 (36-89) years, and 32.6% of the patients had an ASA score of 2. Overall, 30.4% (n=14) of the patients underwent emergent surgery and 69.6% (n=32) underwent elective surgery. Twelve patients underwent subtotal fenestrating cholecystectomy and 34 patients underwent subtotal reconstituting cholecystectomy. Four patients died postoperatively during hospitalisation (Table 1).

**Table 1:** Characteristics of the study group. ASA: American Society of Anaesthesiologists.

				Number (n)	Percentage (%)
Gender	Male			22	47.8
	Female			24	52.2
	Total			46	100.0
ASA score	1			12	26.1
	2			15	32.6
	3			14	30.4
	4			5	10.9
	Total			46	100.0
Timing of surgery			Emergency	14	30.4
			Elective	32	69.6
			Total	46	100.0
Type of the subtotal cholecystectomy		Subtotal fenestrating		12	26.1
		Subtotal reconstituting		34	73.9
		Total		46	100.0
Mortality	Exist			4	8.7
	None			42	91.3
	Total			46	100.0

The comorbidities of the patient group included hypertension as the most common comorbidity (10.9%), followed by cerebrovascular events (CVEs) (8.7%). Wound issues were postoperative complications in 17.4% of the patients, whereas 10.9% (n=5) had a temporary biliary fistula, which resolved spontaneously within postoperative 0-1 month. Two of those patients had undergone the fenestrating type of surgery, and the other three had undergone the reconstituting type of subtotal cholecystectomy. Only one patient who underwent reconstituting cholecystectomy had early high-output biliary drainage (i.e., bile leakage of more than 500 mL) in the early postoperative period and required a second operation. Incisional hernia occurred in 4.3% of the patients who underwent open surgery during the follow-up period (Table 2).

**Table 2:** Postoperative complications. MI: myocardial infarction; CVE: cerebrovascular event.

		Number (n)	Percentage (%)
Postoperative complications	Biloma	1	2.2
	Evisceration and pneumonia	1	2.2
	Fistula	5	10.9
	Incisional hernia	2	4.3
	Pneumonia and pulmonary embolism	1	2.2
	Postoperative MI	1	2.2
	Postoperative CVE	1	2.2
	Wound infection	8	17.4
	Wound infection and pulmonary embolism	1	2.2
	None	25	54.3
	Total	46	100.0

Patients who had postoperative complications had significantly higher co-morbid conditions (78%; p=0.000). However, the timing of the surgery (emergency or elective setting) did not statistically differ in terms of complications (p=0.192). The type of subtotal cholecystectomy also had no statistically significant effect on complications (p=0.409) (52.2% and 45%, respectively; p>0.05) (Table 3).

**Table 3:** Statistical relation of postoperative complications with co-morbidity, timing of surgery, and operation type.

	Postoperative complications	
	Correlation coefficient	p-value
Co-morbidity	0.775	0.000
Timing of surgery	0.519	0.192
Operation type	0.450	0.409

Table 4 has shown the postoperative complications according to the type of subtotal cholecystectomy. 

**Table 4:** Postoperative complications according to the type of the subtotal cholecystectomy. MI: myocardial infarction.

		Postoperative complications										
		Biloma	Evisceration and pneumonia	Fistula	Incisional hernia	Pneumonia and pulmonary embolism	Postoperative MI	Postoperative stroke	Wound infection	Wound infection + pulmonary embolism	None	Total
Surgery type	Subtotal fenestrating cholecystectomy	0	1 (8.3%)	2 (16.7%)	0	0	1 (8.3%)	0	3 (25%)	0	5 (41.7%)	12 (100%)
	Subtotal reconstitutional cholecystectomy	1 (2.9%)	0	3 (8.8%)	2 (5.9%)	1 (2.9%)	0	1 (2.9%)	5 (14.7%)	1 (2.9%)	20 (58.8%)	34 (100%)
	Total	1 (2.2%)	1 (2.2%)	5 (10.9%)	2 (4.3%)	1 (2.2%)	1 (2.2%)	1 (2.2%)	8 (17.4%)	1 (2.2%)	25 (54.3%)	46 (100%)

Patient mortality showed a statistically significant correlation with the patient's comorbidities, the timing of the surgery, and the type of the procedure (p<0.05). The occurrence of postoperative complications also showed a statistically significant correlation with mortality (p<0.05) (Table 5).

**Table 5:** Statistical relation of mortality with co-morbidity, timing of the surgery, surgery type, and postoperative complications.

	Mortality	
	Correlation coefficient	p-value
Co-morbidity	0.929	0.001
Timing of surgery	0.299	0.043
Surgery type	0.344	0.020
Postoperative complication	0.872	0.000

Four (8.7%) of the 46 patients died postoperatively (three had undergone the fenestrating type and one the reconstituting type operation). The deceased patients had a mean age of 81 years and multiple comorbidities (hypertension, diabetes mellitus, coronary heart disease, and congestive heart failure). Three of them underwent emergency surgery, and one underwent elective surgery. Among the causes of mortality, only one patient had mortality related to a wound complication; the other three patients had mortality related to pulmonary causes. Two of them had pneumonia, and one had a massive pulmonary embolism (Table 6).

**Table 6:** Mortality according to the type of the subtotal cholecystectomy.

		Mortality		
		Exist	None	Total
Type of subtotal cholecystectomy	Subtotal fenestrating cholecystectomy	3 (25%)	9 (75%)	12 (100%)
	Subtotal reconstituting cholecystectomy	1 (2.9%)	33 (97.1%)	34 (100%)
	Total	4 (8.7%)	42 (91.3%)	46 (100%)

## Discussion

In this study, the type of subtotal cholecystectomy did not result in any statistical difference in terms of postoperative complications. However, postoperative complications showed a statistically high correlation with co-morbid conditions. Hence, significantly higher mortality rates were found to be correlated with the patients' comorbidities, the timing of the surgery and the type of the procedure.

Our results showed that patients who underwent subtotal cholecystectomy tended to be middle-aged, with a nearly equal gender distribution. Half of the patients had ASA scores of three and four, and only 28.3% (n=13) had no documented comorbidity. As stated in the literature, the subtotal cholecystectomy patients had higher attributable risks than the regular cholecystectomy patients in terms of age and comorbidities [8]. An interpretation may be that patients who underwent subtotal cholecystectomy presented with preoperative risks. Performing a subtotal cholecystectomy is a choice made intraoperatively. However, these patients have medical drawbacks that are difficult to manage in the perioperative setting. The study results also revealed that the timing of the surgery did not show statistically significant differences for complications (p=0.192), in agreement with the literature [9].

Laparoscopic cholecystectomy is the gold standard procedure and has well-established benefits over open surgery. However, it has a two-fold risk of biliary injury, which is very difficult to deal with if it occurs during surgery or postoperatively. These injuries are usually associated with severe inflammation in acute or chronic settings when dissection of the hepatocystic triangle is challenging and a CVS cannot be assured. In these cases, for the surgeon to minimise the risk of bile duct injury, a safe bail-out technique is the optimal choice. One of the five bail-out techniques is subtotal cholecystectomy. Other techniques for difficult cholecystectomy cases are as follows: termination of the entire procedure, conversion to open surgery, tube cholecystostomy, and fundus first cholecystectomy. Using these techniques may be done separately or in combination to diminish concerns regarding cholecystectomy [10].

"Difficult gallbladder surgery" cases are mainly characterised by severe inflammation and distortion of the normal anatomy, and the procedure itself has higher surgical risks than standard cholecystectomies [11]. For these cases, the damage control approach to surgery for acute trauma may offer new insights, offering subtotal cholecystectomy as part of this concept. The benefits of damage control or salvage surgery for severe non-trauma peritonitis patients have recently been addressed [12]. If severe inflammation and difficult dissection are experienced during surgery, subtotal cholecystectomy is an alternate way to reduce local peritonitis with minimal injury [13]. Traditional surgical concepts for maximising surgical exploration through conversion to open surgery are not integral parts of the subtotal "damage-controlled surgery" approach. Surgeons are more familiar with the anatomical perspective of laparoscopic cholecystectomy [14]. Furthermore, conversion to open surgery does not provide any additional benefits for complicated gallbladder surgery [15].

Contrary to the literature that supports laparoscopic surgery as a preferred method over open surgery, all study cases were converted to open surgery before the subtotal cholecystectomy. However, conversion to open surgery in our cases did not improve the anatomical identification of the hepatocystic triangle enough to enable a CVS. Surgeons have gradually accepted the laparoscopic procedure for complicated cases, decreasing the conversion rates to open surgery [16].

In our study group, 12 patients had subtotal fenestrating cholecystectomy and 34 had subtotal reconstituting cholecystectomy. Each of the two techniques has its advantages and drawbacks. After a fenestrating subtotal cholecystectomy, the risk is higher for a significant bile leak that is managed by drains (or endoscopically if needed). This leak may lead to more wound infections and an extended hospital stay. It is more common to see neostone formation and recurrent biliary disease after reconstituting procedures than after fenestrating procedures. While the completion rates were higher for fenestrating cholecystectomy in a published series, retrospective analysis of the reconstituting technique revealed no long-term recurrent symptoms in six cases [17].

Analysis of long-term data from large patient cohorts in the future may clarify the inconsistent data in the literature. The surgeon's expertise and the intraoperative characteristics of the case will determine the optimal procedure. If severe inflammation or fibrosis tissue is present, then closing the remnant is difficult with a reconstituting type [18]. The common principle in the two techniques may be leaving a minimum of inflammatory tissue with a good washout of the gallbladder lumen to extract all the biliary stones.

The two main complications occurring after cholecystectomy are bleeding from the surgical site and biliary injury [19]. We did not experience any pressing bleeding events.

Postoperative biliary leakage occurred in six of our patients who required postoperative endoscopic retrograde cholangiopancreatography (ERCP). Biliary drainage ceased spontaneously in five of the patients. A single patient had to undergo reoperation due to biloma and leakage from the Luschka's duct after a reported suture in the liver bed that resolved. Two of the patients who underwent the fenestrating type of operation and three of those who underwent the reconstituting type had a temporary biliary fistula. The patient who required a reoperation had undergone a subtotal reconstituting cholecystectomy.

Biliary duct injury still raises concerns after cholecystectomy. Subtotal and total cholecystectomy produce similar bile leakage rates. However, the aetiologies of the bile leakages differ. A biliary fistula after subtotal cholecystectomy mainly causes leaks arising from the cystic duct stump and Luschka ducts. The main reasons for subtotal cholecystectomy are to prevent biliary injuries leading to the biliary tree’s discontinuation. These injuries cause significant morbidity and require advanced surgeries. Patients with the higher grades of difficult gallbladders had rates of main duct biliary injury eight times higher than those having lower grades of difficult gallbladders. Our study did not have any pressing biliary injuries that required advanced hepatobiliary surgery. We tend to conduct early ERCP in our patients with biliary leakage, but adequate drainage of the biliary content may postpone this for 14 days after surgery in clinically stable patients. In our study, bile leakage rates did not differ according to the procedure types, contrary to literature reports showing higher bile leakage after fenestrating subtotal cholecystectomy. Patients who underwent reconstituting subtotal cholecystectomy also had lower complication rates in terms of subhepatic collection, conversion, postoperative ERCP, and reoperation needs [6,20].

In general, 54.3% of the patients had no postoperative complications, but 17.4% had wound-related complications. In the literature, wound infections were reported in 2.6% of the patients, while 43.8% of the patients were open subtotal cholecystectomy cases. In our series, 40.6% of the cases that underwent conversion to open surgery had wound infections. Only 15.6% of the patients who underwent laparoscopic subtotal cholecystectomy had wound infections [9]. The high infection rate in our patients may be related to the conversion to open surgery before performing the subtotal cholecystectomy.

Our study results revealed statistically significant correlations between patient mortality and the comorbidities in our patients, the timing of the surgery, and the type of the procedure (p<0.05). The occurrence of postoperative complications also had a statistically significant correlation with mortality (p<0.05).

Four patients in the study group died during hospitalisation (three with the fenestrating operation and one with the reconstituting type of operation). The mean age was 81 years, and all four had multiple comorbidities. Three of them underwent emergency operations, and one had elective surgery. Among the causes of mortality, only one patient had mortality related to a wound complication. The mortality of the other three patients was related to pulmonary causes (two had pneumonia and one had a massive pulmonary embolism). Although mortality showed significant relationships with comorbidity, the timing of the surgery, and the type of procedure, we do not see this as having clinical significance when the patient's volume is too low to interpret the results as general. Patients who are at high risk during any emergency surgery may have increased rates of complications and, consequently, increased absolute mortality.

Our study is one of the few studies that has analysed subtotal cholecystectomy for its postoperative complications and mortality. Comorbidities, the timing of the surgery, and the type of the procedure were all analysed. Statistics revealed relationships between these entities and complications and mortality. We previously performed mainly the reconstituting type, but lately we have favoured fenestrating subtotal cholecystectomy. We think that adopting an appropriate technique and choosing it for the necessary cases may be critical for success in these operations.

This study has several limitations, including its retrospective design and restriction to a single centre. The small number of patients in a five-year postoperative period may not be sufficient to evaluate the exact postoperative complications ratio in the long term. Some patients may not have presented at our institute due to complaints related to the surgery, such as recurrent biliary stone formation. All cases in the study underwent conversion to open surgery, which may inadvertently result in high postoperative complications.

## Conclusions

Subtotal cholecystectomy prevents major biliary complications after cholecystectomy. Usually, the administration of subtotal cholecystectomy is in high-risk patients who have comorbidities. Postoperative complications and mortality may occur. The intraoperative characteristics and the surgeon’s expertise will determine the choice between the two subtotal techniques.
